# Prevalence of measured and reported multimorbidity in a representative sample of the Swiss population

**DOI:** 10.1186/s12889-015-1515-x

**Published:** 2015-02-19

**Authors:** Basile Pache, Peter Vollenweider, Gérard Waeber, Pedro Marques-Vidal

**Affiliations:** Department of medicine, Internal Medicine, University Hospital of Lausanne (CHUV), Rue du Bugnon 46, 1011 Lausanne, Switzerland

**Keywords:** Multimorbidity, Population, Prevalence, Self-report, Switzerland

## Abstract

**Background:**

Little is known on the prevalence of multimorbidity (MM) in the general population. We aimed to assess the prevalence of MM using measured or self-reported data in the Swiss population.

**Methods:**

Cross-sectional, population-based study conducted between 2003 and 2006 in the city of Lausanne, Switzerland, and including 3714 participants (1967 women) aged 35 to 75 years. Clinical evaluation was conducted by thoroughly trained nurses or medical assistants and the psychiatric evaluation by psychologists or psychiatrists. For psychiatric conditions, two definitions were used: either based on the participant’s statements, or on psychiatric evaluation. MM was defined as presenting ≥2 morbidities out of a list of 27 (self-reported – definition A, or measured – definition B) or as the Functional Comorbidity Index (FCI) using measured data – definition C.

**Results:**

The overall prevalence and (95% confidence interval) of MM was 34.8% (33.3%-36.4%), 56.3% (54.6%-57.9%) and 22.7% (21.4%-24.1%) for definitions A, B and C, respectively. Prevalence of MM was higher in women (40.2%, 61.7% and 27.1% for definitions A, B and C, respectively, *vs*. 28.7%, 50.1% and 17.9% in men, p < 0.001); Swiss nationals (37.1%, 58.8% and 24.8% for definitions A, B and C, respectively, *vs*. 31.4%, 52.3% and 19.7% in foreigners, all p < 0.001); elderly (>65 years: 67.0%, 70.0% and 36.7% for definitions A, B and C, respectively, *vs*. 23.6%, 50.2% and 13.8% for participants <45 years, p < 0.001); participants with lower educational level; former smokers and obese participants. Multivariate analysis confirmed most of these associations: odds ratio (95% Confidence interval) 0.55 (0.47-0.64), 0.61 (0.53-0.71) and 0.51 (0.42-0.61) for men relative to women for definitions A, B and C, respectively; 1.27 (1.09-1.49), 1.29 (1.11-1.49) and 1.41 (1.17-1.71) for Swiss nationals relative to foreigners, for definitions A, B and C, respectively. Conversely, no difference was found for educational level for definitions A and B and abdominally obese participants for all definitions.

**Conclusions:**

Prevalence of MM is high in the Lausanne population, and varies according to the definition or the data collection method.

**Electronic supplementary material:**

The online version of this article (doi:10.1186/s12889-015-1515-x) contains supplementary material, which is available to authorized users.

## Background

Multimorbidity (MM) is defined as the combined presence of several chronic pathologies in a subject [1]. MM increases with age [2] and has been associated with a decrease in quality of life [3,4], with increased health care utilization and cost [4,5] and with increased mortality [4], although this latter statement has been challenged [6].

The definition of MM is far from consensual. A review on the measures of MM in the clinical practice setting listed 17 different measures, from disease counts to more sophisticated indexes [7]. Another review collecting data from 12 countries reported that the number of health conditions analysed per study ranged from 5 to 335 [8]. This lack of standardization leads to a considerable difference in prevalence levels [9], which can range from less than 5% to over 95% [8,10]. This lack of standardization also leads to different associations with health outcomes [11].

The prevalence of MM has been extensively examined among hospitalized patients or in clinical practices [5,7,8,10,12-14]. Still, few studies have assessed the prevalence of MM in Switzerland [14] and, to our knowledge, none has ever assessed the prevalence of MM in the general population.

Therefore, the objective of this study was to assess the prevalence of MM in a representative sample of the Swiss population aged 35 to 75 years, using different MM definitions and either reported or objectively assessed morbidity.

## Methods

### The Cohorte Lausannoise (CoLaus) study

The CoLaus study is a population-based study assessing the clinical, biological and genetic determinants of cardiovascular disease in the city of Lausanne, Switzerland. Complete details of the sampling and clinical assessments are provided elsewhere [15]. The study was approved by the Institutional Ethics Committee of the University of Lausanne and all participants provided written informed consent. The initial recruitment took place between June 2003 and May 2006 and enrolled 6,733 participants (3,544 women) aged 35-75 years; participation rate was 41%. A subset of 3,712 participants also had an extensive psychiatric evaluation [16]. Clinical evaluation was conducted either by nurses or medical assistants who were trained over a month period; psychiatric evaluation was conducted either by psychologists or psychiatrists, who were trained over a two-months period [16].

### Clinical and anthropometric data

Educational level was categorized as primary, apprenticeship, secondary school and university. Smoking status was defined as never, former and current. Receiving social help was assessed with the question: “Do you receive social help?”. Because all individuals residing in Switzerland receive financial compensation when they retire, the response to this variable is not informative beyond the retirement age. Therefore, men older than 65 years and women older than 63 or 64 (depending on sampling year, as retirement age changed for women during the survey period) years were not considered as receiving social help.

Body weight, height and waist circumference (WC) were measured using standard procedures [15]. Body mass index (BMI) was defined as weight(kg)/height(m)^2^. Overweight was defined as 25 ≤ BMI < 30 kg/m^2^ and obesity as BMI ≥ 30 kg/m^2^. Abdominal obesity was defined as a waist circumference ≥102 cm for men and ≥88 cm for women.

### Multimorbidity

Multimorbidity was defined according to two sets of criteria [12,17]. The first one [12] was chosen because of the large sample size it was built upon and availability of the criteria in our study. For non-psychiatric conditions, presence was established based either on self-report or on the existence of a drug treatment. For psychiatric conditions, two possibilities were assessed: either based on the participant’s statements, or on psychiatric evaluation. This was decided as most studies on MM rely on self-report of psychiatric diseases and seldom use a comprehensive examination as in this study. As both self-reported and objectively measured data were available, two definitions were considered: definition A included only self-reported morbidities, while in definition B all available objectively measured morbidities (either diagnosed or assessed by an existing medication) replaced the self-reported ones. These definitions are of interest in a public health setting, but are difficult to implement in clinical practice as they require the collection of a large number of conditions.

Finally, a third definition (named C) was based on one of the most used scores to assess MM, the Functional Comorbidity Index (FCI) [17]. The criteria used for each definition are indicated in the Additional file 1: Table S1. This definition is based on a smaller number of conditions and is easier to implement in clinical practice.

For each participant, the number of conditions present according to each definition (see Additional file 1: Table S1) was counted. For each definition, MM was defined if a participant had more than 2 conditions, as performed by others [12].

### Statistical analysis

Statistical analyses were conducted using Stata v.13 (Stata Corp, College Station, TX, USA). Descriptive results were expressed as average ± standard deviation or as number of participants and (percentage). Bivariate comparisons were performed using Student’s t-test or one-way analysis of variance (ANOVA) for quantitative data and chi-square for categorical data. Associations between MM scores were assessed using Spearman rank correlation. Multivariate analysis was conducted using multivariate ANOVA for number of pathologies and using logistic regression for multimorbidity status (yes/no). Results of the logistic regression were expressed as odds ratio and (95% confidence interval). Statistical significance was assessed for p < 0.05.

## Results

### Characteristics of the participants

The characteristics of the sample according to gender are summarized in Table 1. Women were older, less well educated, more frequently non-smokers, had a lower BMI and a lower prevalence of overweight and obesity. No differences were found between genders regarding social help or being born in Switzerland (Table 1).

**Table 1:** **Clinical characteristics of the participants, by gender**

	**Women (N = 1967)**	**Men (N = 1747)**	**p-value**
**Age** (years)	50.0 ± 8.8	49.1 ± 8.8	0.004
**Educational level** (%)			
Primary	368 (18.7)	246 (14.1)	
Apprenticeship	672 (34.2)	596 (34.1)	<0.001
High school/college	559 (28.4)	442 (25.3)	
University	368 (18.7)	463 (26.5)	
**Social help** (%)	307 (15.6)	241 (13.8)	0.12
**Born in Switzerland** (%)	1210 (61.6)	1035 (59.2)	0.15
**Smoking status** (%)			
Never	855 (43.5)	604 (34.6)	
Former	566 (28.8)	627 (35.9)	0.001
Current	544 (27.7)	516 (29.5)	
**BMI** (kg/m^2^)	24.8 ± 4.9	26.3 ± 3.8	<0.001
**BMI categories** (%)			
Normal	1206 (61.4)	708 (40.5)	
Overweight	516 (26.3)	787 (45.1)	<0.001
Obese	243 (12.4)	252 (14.4)	
**Waist** (cm)	82.1 ± 12.4	94.3 ± 10.9	<0.001
**Abdominal obesity** (%)	564 (28.7)	379 (21.7)	<0.001

BMI, body mass index. Results are expressed as number of participants (percentage) or as average ± standard deviation. Statistical analysis by chi-square or Student’s t-test.

### Prevalence of multimorbidity

The prevalence of MM according to the three definitions considered and with several characteristics of the participants is summarized in Table 2.

**Table 2:** **Prevalence of multimorbidity according to three different definitions and the participants’ characteristics**

	**Definition A**	**Definition B**	**Definition C**
**All**	1294 (34.8)	2089 (56.3)	845 (22.7)
**Gender**			
Woman	790 (40.2)	1213 (61.7)	533 (27.1)
Man	502 (28.7)	876 (50.1)	312 (17.9)
p-value between groups	<0.001	<0.001	<0.001
**Age group**			
35-44	314 (23.6)	671 (50.2)	184 (13.8)
45-54	418 (33.9)	697 (56.7)	275 (22.4)
55-64	499 (47.1)	658 (62.3)	353 (33.4)
65+	61 (67.0)	63 (70.0)	33 (36.7)
p-value between groups	<0.001	<0.001	<0.001
**Education**			
Primary	259 (42.2)	357 (58.1)	191 (31.1)
Apprenticeship	480 (37.9)	747 (58.9)	328 (25.9)
High school/college	319 (31.9)	555 (55.4)	203 (20.3)
University	236 (28.4)	430 (51.7)	123 (14.8)
p-value between groups	<0.001	<0.01	<0.001
**Receiving social help**			
No	1004 (31.7)	1708 (54.0)	625 (19.7)
Yes	290 (52.9)	381 (69.5)	220 (40.2)
p-value between groups	<0.001	<0.001	<0.001
**Born in Switzerland**			
No	460 (31.4)	768 (52.3)	289 (19.7)
Yes	832 (37.1)	1321 (58.8)	556 (24.8)
p-value between groups	<0.001	<0.001	<0.001
**Smoking**			
Never	440 (30.2)	714 (48.9)	288 (19.7)
Former	462 (38.7)	721 (60.4)	304 (25.5)
Current	390 (36.8)	654 (61.6)	253 (23.9)
p-value between groups	<0.001	<0.001	0.001
**BMI status**			
Normal	560 (29.3)	1054 (55.0)	302 (15.8)
Overweight	470 (36.1)	700 (53.7)	235 (18.0)
Obese	262 (52.9)	335 (67.5)	308 (62.1)
p-value between groups	<0.001	<0.001	<0.001
**Abdominal obesity**			
No	830 (30.0)	1474 (53.2)	435 (15.7)
Yes	464 (49.2)	615 (65.2)	410 (43.4)
p-value between groups	<0.001	<0.001	<0.001

Results are expressed as number of participants (percentage) presenting with the condition. BMI, body mass index. Definitions A and B based on [12]. Definition C based on the Functional Comorbidity Index [17]. For more details, please consult Additional file 1: Table S1. Statistical analysis between groups stratified on the definition of multimorbidity using chi-square.

**Figure 1:**
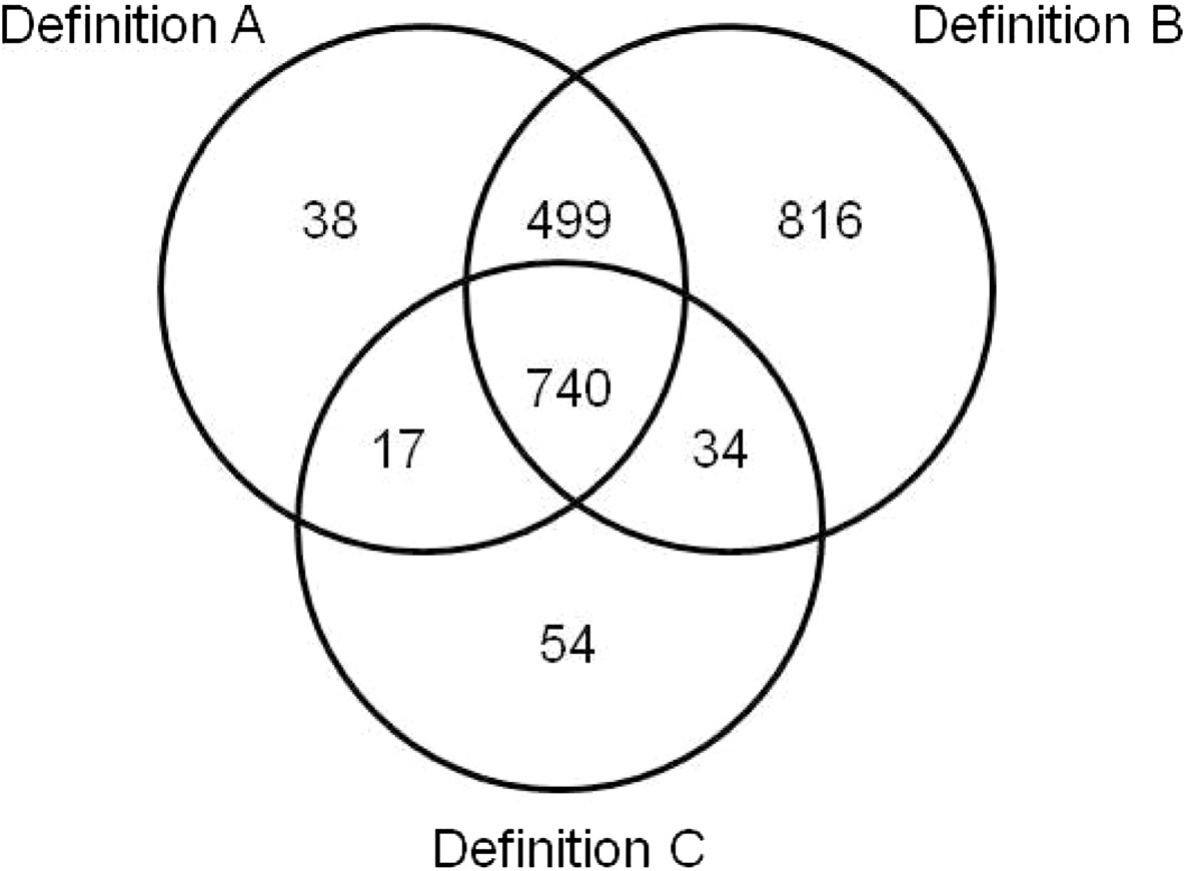
**Venn diagram showing the prevalence of multimorbidity according to the definitions considered.** Definition A, using reported morbidities; definition B, using measured morbidities; definition C, using Functional Comorbidity Index (FCI) criteria.

The overall prevalence of MM using measured morbidities (definition B) was the highest, followed by MM using reported morbidities (definition A) and MM according to FCI (definition C). Significant (p < 0.001) correlations were found between MM scores (Additional file 2: Figures S1-S3), with Spearman r = 0.744 between definitions A and B, r = 0.560 between definitions B and C, and r = 0.765 between definitions A and C. The number of participants diagnosed with MM according to each definition is indicated in Figure 1. Only one third (33.7%) of participants diagnosed with MM was jointly diagnosed by all three definitions considered.

### Determinants of multimorbidity

Irrespective of the definition used, the prevalence of MM was higher in women, among participants receiving social help and among current and former smokers. The prevalence of MM also increased considerably with age and obesity (defined by BMI or increased waist) and with decreasing educational level (Table 2).

The results of the multivariate analysis of the factors associated with each definition of MM are summarized in Table 3. For all definitions of MM considered, men had a lower likelihood of presenting MM than women. Older age, receiving social help, being born in Switzerland, being a former or a current smoker and being obese were significantly and independently associated with a higher likelihood of presenting MM. No effect of educational level was found for definitions A and B of MM, while for definition C a protective effect of education was found. No association was found between abdominal obesity and MM for all three definitions of MM considered.

**Table 3:** **multivariate analysis of factors associated with multimorbidity according to three definitions**

	**Definition A**	**Definition B**	**Definition C**
**Gender**			
Woman	1 (ref.)	1 (ref.)	1 (ref.)
Man	0.55 (0.47 - 0.64)	0.61 (0.53 - 0.71)	0.51 (0.42 - 0.61)
**Age group**			
35-44	1 (ref.)	1 (ref.)	1 (ref.)
45-54	1.59 (1.33 - 1.91)	1.24 (1.05 - 1.45)	1.73 (1.39 - 2.17)
55-64	2.45 (2.03 - 2.95)	1.44 (1.21 - 1.71)	2.52 (2.01 - 3.16)
65+	6.91 (4.28 - 11.1)	2.31 (1.43 - 3.71)	3.36 (2.01 - 5.62)
p-value for trend	<0.001	<0.001	<0.001
**Education**			
Primary	1 (ref.)	1 (ref.)	1 (ref.)
Apprenticeship	0.84 (0.67 - 1.04)	1.00 (0.81 - 1.24)	0.81 (0.63 - 1.05)
High school/college	0.77 (0.61 - 0.96)	0.98 (0.79 - 1.22)	0.76 (0.58 - 0.99)
University	0.82 (0.64 - 1.04)	0.99 (0.79 - 1.24)	0.68 (0.50 - 0.91)
p-value for trend	0.11	0.99	<0.05
**Receiving social help**			
No	1 (ref.)	1 (ref.)	1 (ref.)
Yes	2.40 (1.97 - 2.92)	1.92 (1.56 - 2.35)	2.74 (2.19 - 3.41)
**Born in Switzerland**			
No	1 (ref.)	1 (ref.)	1 (ref.)
Yes	1.27 (1.09 - 1.49)	1.29 (1.11 - 1.49)	1.41 (1.17 - 1.71)
**Smoking**			
Never	1 (ref.)	1 (ref.)	1 (ref.)
Former	1.54 (1.30 - 1.84)	1.69 (1.44 - 1.98)	1.55 (1.26 - 1.91)
Current	1.51 (1.26 - 1.80)	1.79 (1.51 - 2.11)	1.51 (1.21 - 1.87)
p-value for trend	<0.001	<0.001	<0.001
**BMI status**			
Normal	1 (ref.)	1 (ref.)	1 (ref.)
Overweight	1.33 (1.11 - 1.59)	0.94 (0.80 - 1.11)	1.10 (0.88 - 1.38)
Obese	2.18 (1.63 - 2.92)	1.41 (1.06 - 1.88)	7.53 (5.40 - 10.5)
p-value for trend	<0.001	0.16	<0.001
**Abdominal obesity**			
No	1 (ref.)	1 (ref.)	1 (ref.)
Yes	1.16 (0.93 - 1.45)	1.22 (0.98 - 1.51)	1.24 (0.96 - 1.60)

Results are expressed as Odds ratio and (95% confidence interval). BMI, body mass index. Definitions A and B based on [12]. Definition C based on the Functional Comorbidity Index [17]. For more details, please consult Additional file 1: Table S1. Statistical analysis by multivariate logistic regression adjusting simultaneously for all parameters indicated.

## Discussion

To our knowledge, this is one of the few studies that assessed the prevalence of MM using a population-based sample and the first of its kind in Switzerland. Our results show that MM is relatively common in an apparently healthy general population. Our results also show that the prevalence of MM varies significantly according to the criteria used and even according to the data collection method (reported or measured).

### Prevalence of multimorbidity

Prevalence of MM varied considerably according to the criteria used, a finding already reported in the literature [9,11], although this statement has been challenged [14]. Even using the same set of criteria, the prevalence varied considerably when self-reported or measured data was collected, a finding also noted when using data from electronic records or from health surveys [18,19]. A likely explanation is that many subjects are unaware of their status, as it has been shown for cardiovascular risk factors such as hypertension [20] or type 2 diabetes [21]. The lower prevalence of MM according to the FCI might also be due to the fact that the number of criteria is lower than the other definitions. Hence, a condition present in definitions A and B might not be considered as such with the FCI definition. Our results suggest that the prevalence of MM depends on the number of conditions considered, the higher the number the higher the likelihood of being diagnosed with MM. A possible (but not optimal) solution would be to modulate the threshold according to the number of criteria used to facilitate comparison between studies: for instance, MM could be diagnosed if a participant has 30% of all conditions, instead of a fixed number of conditions. Another possibility would be to select different definitions of MM according to the objective of the study [11], but this possibility would limit comparisons to studies with the same aims.

Although significant correlations were found between the number of reported or measured morbidities, still no good agreement was found between definitions, as only one third of participants diagnosed with MM by at least one definition was jointly diagnosed as MM by all three definitions. Our results thus stress the need for a common, standard definition of MM, which will allow comparison between studies.

The high prevalence of MM in our study also raises the question of the adequate management of subjects with MM. Indeed, health care providers are usually trained to manage one disease at the time (single-disease approach), with a further specialization for some conditions. Our results thus question the current status of medical training and of the medical system, and indicate that future medical education should bring back together different branches of medicine, as already postulated [22].

### Determinants of multimorbidity

Women had higher rates of MM than men, and this association was confirmed after multivariate adjustment. These findings are in agreement with some studies [12,23] but not with others [2,14,24]. One explanation is that women are more sensitized to their health status and thus tend to report more conditions (Additional file 1: Table S2), but this should not influence MM defined according to objectively measured data, although the prevalence of objectively assessed psychiatric diseases was higher in women than in men (Additional file 1: Table S2). Overall, our results indicate that MM is more prevalent in Swiss women than in Swiss men, and that this difference is independent from other demographic, clinical or socio-economic characteristics.

Prevalence of MM increased considerably with age, a finding also reported in the literature [2,12,14,23]. This was mainly due to the increase in the overall number of participants with at least one condition (Additional file 2: Figures S4-S6). Indeed, age is related to functional decline and to an increase in the number of morbid conditions. Interestingly, the difference in the prevalence of MM using self-reported and measured data tended narrow with increasing age. This narrowing could be explained by a better awareness or a better diagnosis of the diseases; indeed, it has been shown that a significant percentage of cardiovascular risk factors are undiagnosed [20,21,25]. Overall, our results confirm that age is a strong determinant of MM, and that MM is prevalent even among young adults, a finding also reported elsewhere [26]. More importantly, our results suggest that, in developed countries, the total number of patients with MM will considerably increase in the forthcoming years because of the ageing population, with considerable impact on health care costs [27].

In agreement with other studies [28,29], prevalence of MM was higher among obese participants. The considerable increase of MM as defined by FCI among obese participants is easily explained by the fact that obesity is a criterion for MM according to FCI. Overall, our results reflect the clustering of risk factors and morbidities among obese subjects [29,30], and in future studies it will be of interest the joint trends in obesity and MM, namely in the younger population. Finally, the positive association between abdominal obesity and MM initially found on bivariate analysis became non-significant after multivariate analysis.

Being a former or current smoker was related to a higher prevalence of MM, a finding also reported previously [26]. As for obesity, the most likely explanation is the tobacco-induced increase of multiple morbid conditions. Hence and again, early smoking cessation should be offered to all current smokers in order to decrease their risk of disease and, consequently, of MM.

Low socio economic status, defined by a low education or by receiving social help, was positively associated with MM, although the association between educational level and MM was significant for the FCI definition only. Our results are in agreement with several studies [2,12,31,32] but not with another [33]. Possible explanations include the deleterious effect of working environment together with inadequate health behaviours among low SES groups. Overall, our results suggest that preventive measures should be directed to low SES groups, but such measures are different to implement [34] and their effects are controversial [35].

Swiss citizenship was positively associated with MM. This finding was somewhat unexpected as in Switzerland access to health care is available for all. Further, to our knowledge, no study ever focused on MM in migrants. Thus, reasons for this difference are not straightforward and can only be speculated. One possible explanation would be a higher use of the healthcare system by Swiss nationals, which would increase the likelihood of detecting diseases. Another possible explanation would be better health behaviours of migrants relatively to Swiss nationals [36], but future studies are needed to better assess this point.

### Strengths and limitations

This study has several strengths. It was conducted on an apparently healthy, population-based sample. It also collected data on self-reported and measured morbidities, allowing the comparison of the two data collection methods.

This study has also several limitations. The participation rate (41%) was relatively low, although in line or event higher than other epidemiological studies [37,38]. Hence, a selection bias cannot be ruled out, subjects presenting with morbidities being more prone to refuse to participate. Similarly, some participants might have forgotten to report some diseases or incorrectly reported them. Still, this would lead to an underestimation of the true prevalence of MM within the target population; thus, we believe that our prevalence estimates, although relatively high, are even though rather conservative. Due to its cross-sectional design, no association could be made between MM severity and quality of life or mortality. The ongoing follow-up of the CoLaus cohort will enable assessing the trends in the prevalence of MM and the associations between the different definitions of MM and mortality. Finally, in Switzerland, matching of medical electronic records with information from surveys is not allowed. Thus, it was not possible to confirm the statements of the participants from electronic records. Still, several studies have shown a discrepancy between data collected by surveys and extracted from electronic health records [18,19]: depending on the disease of interest, the prevalence obtained from electronic records could be similar, higher or lower than the prevalence reported in the survey, with further variations according to age and sex [19].

## Conclusion

Prevalence of MM in the general population varies considerably according to the definition used. Increased age, female gender, tobacco smoking, obesity and low socioeconomic status significantly increase the likelihood of MM. The effect of migration awaits further investigation.

## Additional files

Additional file 1: Table S1. Criteria used to define multimorbidity. **Table S2.** Prevalence of the most common morbidities overall and by gender, CoLaus study. Additional file 2: Figure S1. Association between the number of reported and measured conditions. **Figure S2.** Association between the number of reported conditions and the number of conditions according to the Functional Comorbidity Index (FCI). **Figure S3.** Association between the number of measured conditions and the number of conditions according to the Functional Comorbidity Index (FCI). **Figure S4.** Percentage of participants with one or more condition(s), using self-reported conditions. **Figure S5.** Percentage of participants with one or more condition(s), using measured conditions. **Figure S6.** Percentage of participants with one or more condition(s), using Functional Comorbidity Index (FCI) criteria.
