# Supplementary material for: Prevalence of measured and reported multimorbidity in a representative sample of the Swiss population
Source: BMC Public Health. 2015 Feb 19;15:164. doi: 10.1186/s12889-015-1515-x (PMC4336755; doi:10.1186/s12889-015-1515-x)
Supplement: Additional file 1: Table S1. — Criteria used to define multimorbidity. Table S2. Prevalence of the most common morbidities overall and by gender, CoLaus study. [file 12889_2015_1515_MOESM1_ESM.docx]

**Additional file 1: Table S1:** criteria used to define multimorbidity.

| **Pathology** | **Definition A** | **Definition B** | **Definition C** |
| --- | --- | --- | --- |
| **Psychiatric, as diagnosed by the psychiatric evaluation** |  |  |  |
| Alcohol problems | X | X |  |
| Anorexia or bulimia | X | X |  |
| Anxiety |  | X |  |
| Depression |  | X |  |
| Epilepsy |  | X |  |
| Migraine |  | X |  |
| Schizophrenia |  | X |  |
| Substance misuse | X | X |  |
| **Psychiatric, as reported by the participant** |  |  |  |
| Anxiety~~, reported~~ | X |  | X |
| Depression~~, reported~~ | X |  | X |
| Epilepsy~~, reported~~ | X |  |  |
| Migraine~~, reported~~ | X |  |  |
| Schizophrenia~~, reported~~ | X |  |  |
| **Non-psychiatric** |  |  |  |
| **Cancer** (all) | X | X |  |
| **Endocrine** |  |  |  |
| Diabetes | X | X | X |
| Obesity |  |  | X |
| Thyroid disorders | X | X |  |
| **Neurological** |  |  |  |
| Multiple sclerosis | X | X |  |
| Painful condition | X | X |  |
| Parkinson's disease | X | X |  |
| Peripheral neuropathy | X | X |  |
| **Eye and ear** |  |  |  |
| Glaucoma | X | X | X |
| Impaired vision | X | X | X |
| Hearing loss | X | X |  |
| **Cardiovascular** |  |  |  |
| Arrhythmia | X | X |  |
| Congestive heart failure | X | X | X |
| Coronary heart disease | X | X | X |
| Hypertension | X | X |  |
| Peripheral artery disease | X | X | X |
| Stroke | X | X | X |
| **Pulmonary** |  |  |  |
| Asthma |  | X | X |
| Asthma, reported | X |  |  |
| Chronic obstructive pulmonary disease | X | X | X |
| **Gastro-intestinal** |  |  |  |
| Chronic liver disease | X | X |  |
| Hepatitis | X | X |  |
| Upper gastroesophageal disease | X | X | X |
| Constipation | X | X |  |
| Dyspepsia | X | X | X |
| Irritable bowel syndrome | X | X |  |
| Inflammatory bowel disease | X | X |  |
| **Skin** |  |  |  |
| Psoriasis, if treated |  | X |  |
| Psoriasis, if reported by the patient | X |  |  |
| **Bone and joints** |  |  |  |
| Arthritis | X | X | X |
| Degenerative disk disease | X | X | X |
| Osteoporosis | X | X | X |

Definitions A and B based on ([7](#_ENREF_7)). Definition C based on the Functional Comorbidity Index ([14](#_ENREF_14)). Multimorbidity was defined as the presence of ≥2 criteria.

**Additional file 1: Table S2**: Prevalence of the most common morbidities overall and by gender, CoLaus study.

| **Pathology** | **Women (n=1967)** | **Men**  **(n=1747)** | **All**  **(n=3714)** |
| --- | --- | --- | --- |
| Depression | 1060 (53.9) | 564 (32.3) | 1624 (43.7) |
| Depression, reported | 465 (23.6) | 199 (11.4) | 664 (17.9) |
| Anxiety | 591 (30.1) | 344 (19.7) | 935 (25.2) |
| Anxiety, reported | 211 (10.7) | 114 (6.5) | 325 (8.8) |
| Hypertension | 366 (18.6) | 443 (25.4) | 809 (21.8) |
| Arthritis | 493 (25.1) | 280 (16.0) | 773 (20.8) |
| Migraine | 392 (19.9) | 170 (9.7) | 562 (15.1) |
| Migraine, reported | 401 (20.4) | 161 (9.2) | 562 (15.1) |
| Obesity | 244 (12.4) | 252 (14.4) | 496 (13.4) |
| Alcohol problems | 97 (4.9) | 339 (19.4) | 436 (11.7) |
| Asthma, reported | 219 (11.1) | 144 (8.2) | 363 (9.8) |
| Asthma | 62 (3.2) | 43 (2.5) | 105 (2.8) |
| Substance misuse | 71 (3.6) | 159 (9.1) | 230 (6.2) |
| Arrhythmia | 137 (7.0) | 81 (4.6) | 218 (5.9) |
| Psoriasis, reported | 79 (4.0) | 98 (5.6) | 177 (4.8) |
| Psoriasis | 2 (0.1) | 8 (0.5) | 10 (0.3) |
| Diabetes | 55 (2.8) | 97 (5.6) | 152 (4.1) |
| Thyroid disorders | 124 (6.3) | 18 (1.0) | 142 (3.8) |
| Chronic obstructive pulmonary disease | 74 (3.8) | 56 (3.2) | 130 (3.5) |
| Schizophrenia | 49 (2.5) | 45 (2.6) | 94 (2.5) |
| Schizophrenia, reported | 20 (1.0) | 22 (1.3) | 42 (1.1) |
| Coronary heart disease | 27 (1.4) | 57 (3.3) | 84 (2.3) |
| Cancer | 70 (3.6) | 15 (0.9) | 85 (2.3) |
| Anorexia or bulimia | 67 (3.4) | 6 (0.3) | 73 (2.0) |
| Peripheral artery disease | 37 (1.9) | 26 (1.5) | 63 (1.7) |
| Hepatitis | 22 (1.1) | 32 (1.8) | 54 (1.5) |
| Epilepsy, reported | 26 (1.3) | 21 (1.2) | 47 (1.3) |
| Epilepsy | 17 (0.9) | 25 (1.4) | 42 (1.1) |
| Stroke | 23 (1.2) | 21 (1.2) | 44 (1.2) |
| Upper gastroesophageal disease | 17 (0.9) | 15 (0.9) | 32 (0.9) |
| Constipation | 19 (1.0) | 10 (0.6) | 29 (0.8) |
| Irritable bowel syndrome | 19 (1.0) | 7 (0.4) | 26 (0.7) |
| Inflammatory bowel disease | 13 (0.7) | 12 (0.7) | 25 (0.7) |
| Painful condition | 7 (0.4) | 17 (1.0) | 24 (0.7) |
| Dyspepsia | 16 (0.8) | 6 (0.3) | 22 (0.6) |
| Glaucoma | 10 (0.5) | 8 (0.5) | 18 (0.5) |
| Degenerative disk disease | 10 (0.5) | 8 (0.5) | 18 (0.5) |
| Impaired vision | 3 (0.2) | 8 (0.5) | 11 (0.3) |
| Peripheral neuropathy | 5 (0.3) | 4 (0.2) | 9 (0.2) |
| Chronic liver disease | 7 (0.4) | 2 (0.1) | 9 (0.2) |
| Congestive heart failure | 2 (0.1) | 3 (0.2) | 5 (0.1) |
| Hearing loss | 2 (0.1) | 2 (0.1) | 4 (0.1) |
| Multiple sclerosis | 2 (0.1) | 2 (0.1) | 4 (0.1) |
| Parkinson's disease | 1 (0.1) | 2 (0.1) | 3 (0.1) |

Results are expressed as number of participants and (percentage) and by descending order of overall frequency.
