# Supplementary material for: Prevalence of measured and reported multimorbidity in a representative sample of the Swiss population
Source: BMC Public Health. 2015 Feb 19;15:164. doi: 10.1186/s12889-015-1515-x (PMC4336755; doi:10.1186/s12889-015-1515-x)

**Additional file 2: Figure S1**: association between the number of reported and measured conditions. The marker size is proportional to the number of participants.


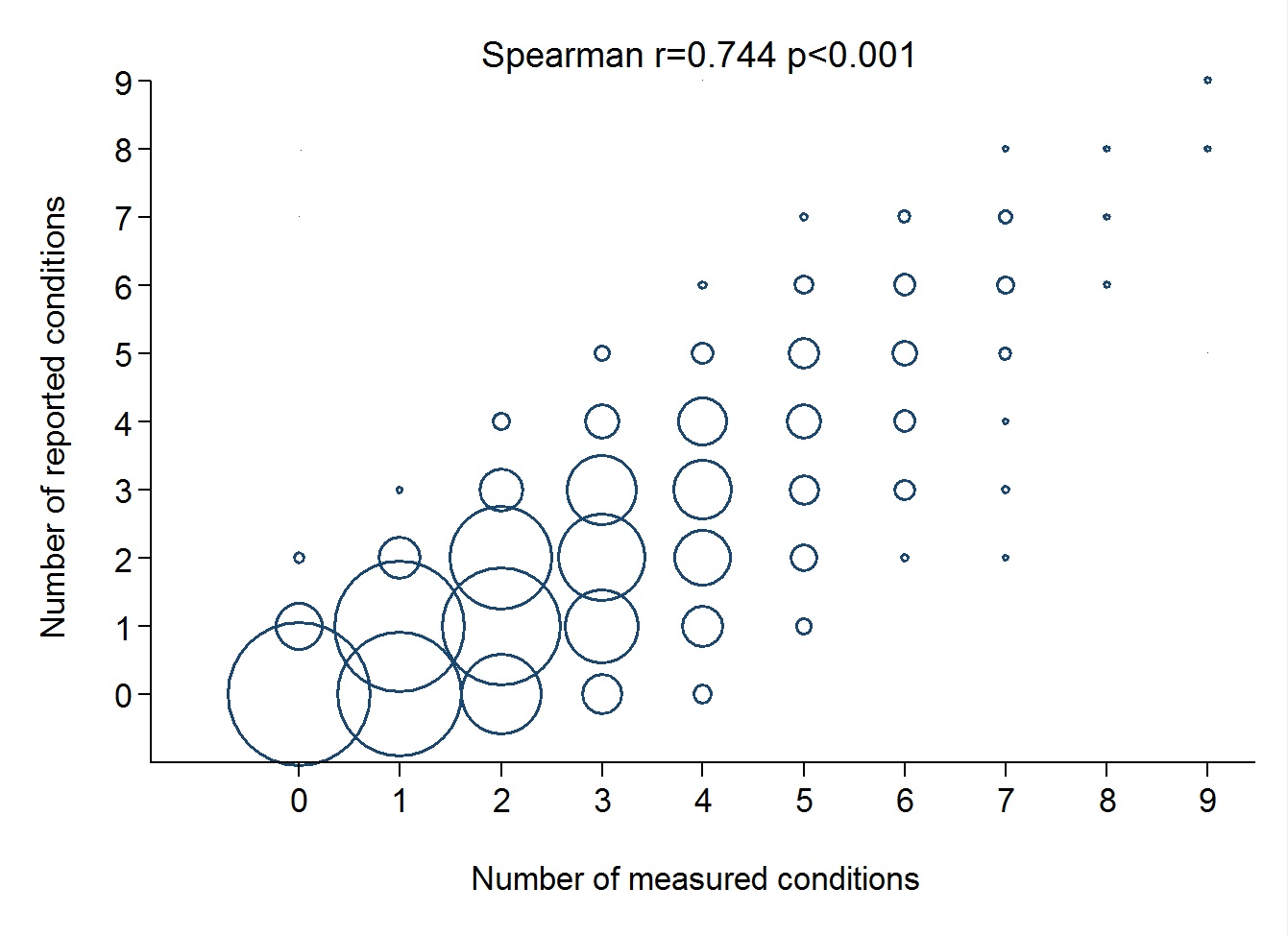


**Additional file 2: Figure S2**: association between the number of reported conditions and the number of conditions according to the Functional Comorbidity Index (FCI). The marker size is proportional to the number of participants.


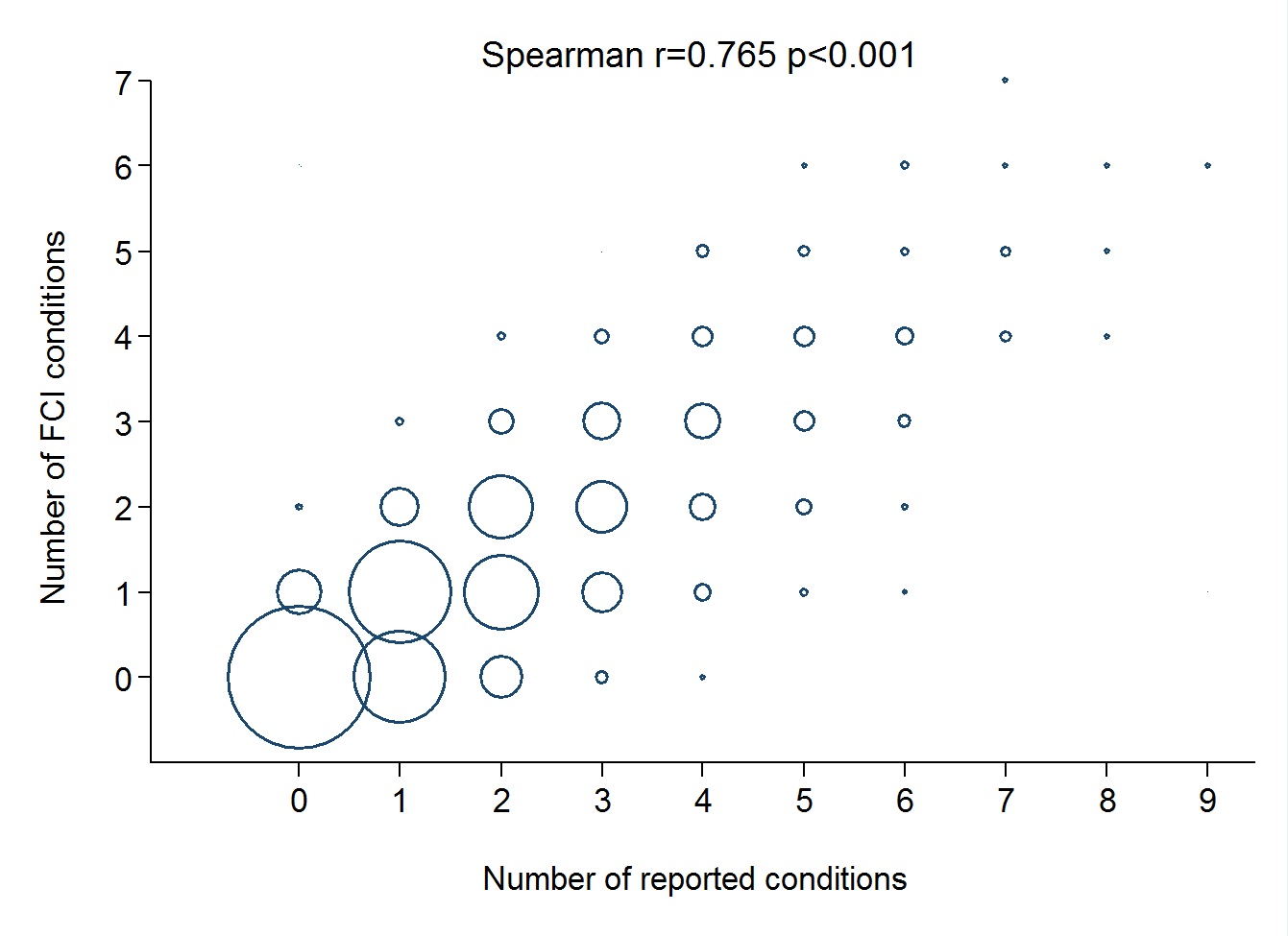


**Additional file 2: Figure S3**: association between the number of measured conditions and the number of conditions according to the Functional Comorbidity Index (FCI). The marker size is proportional to the number of participants.


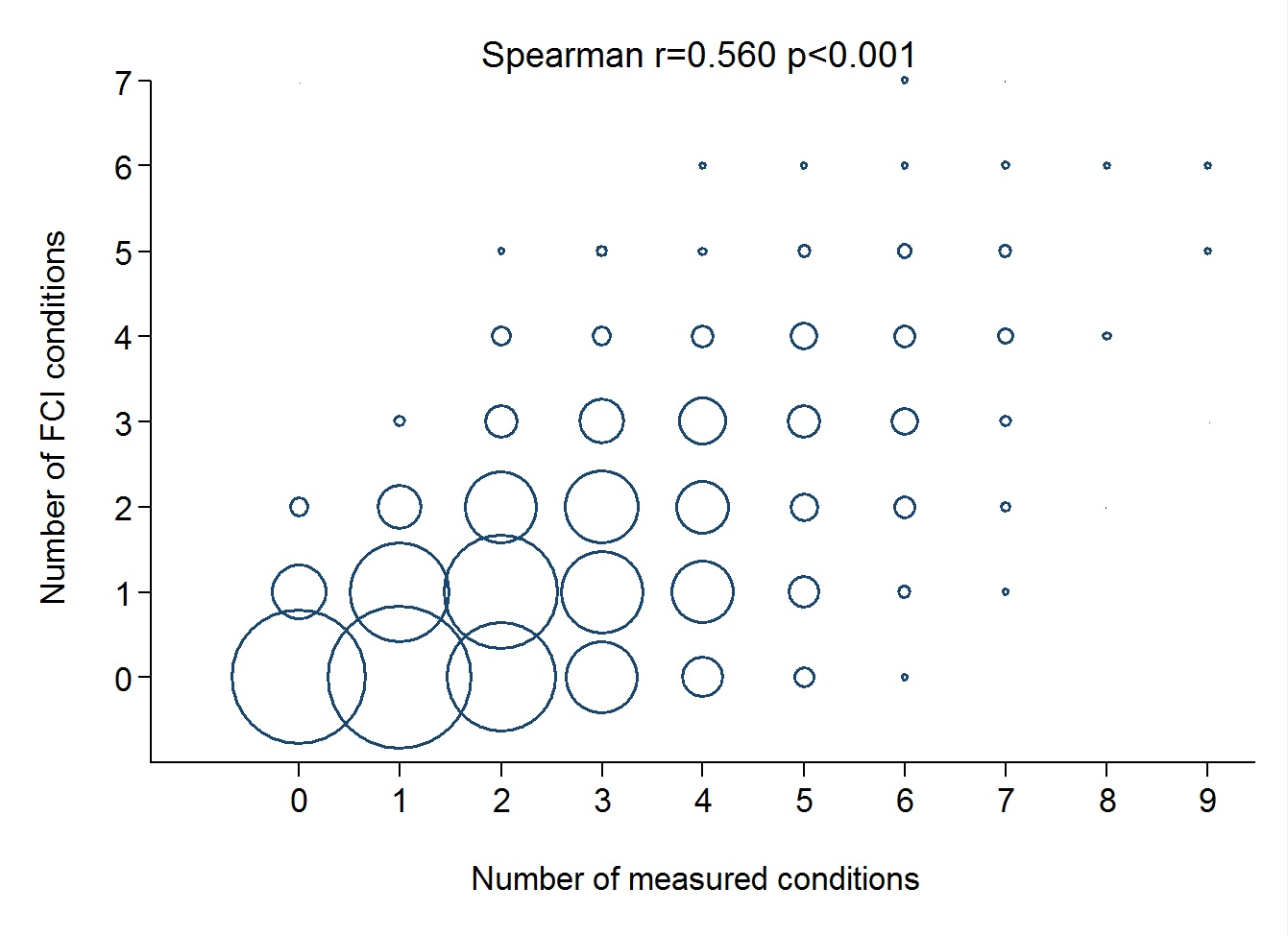


**Additional file 2: Figure S4**: percentage of participants with one or more condition(s), definition A.


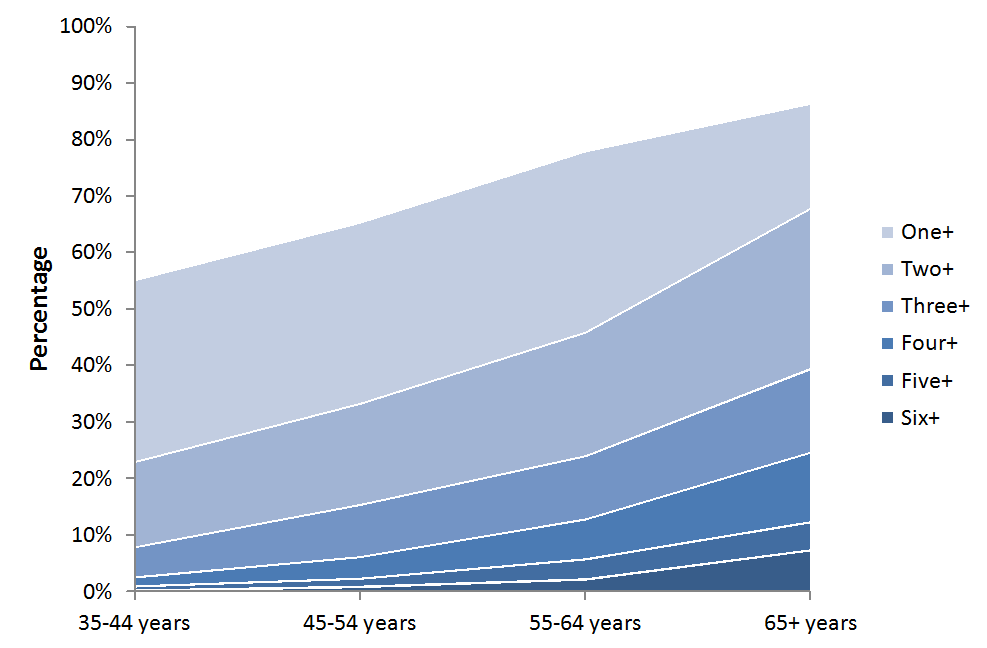


**Additional file 2: Figure S5**: percentage of participants with one or more condition(s), definition B.


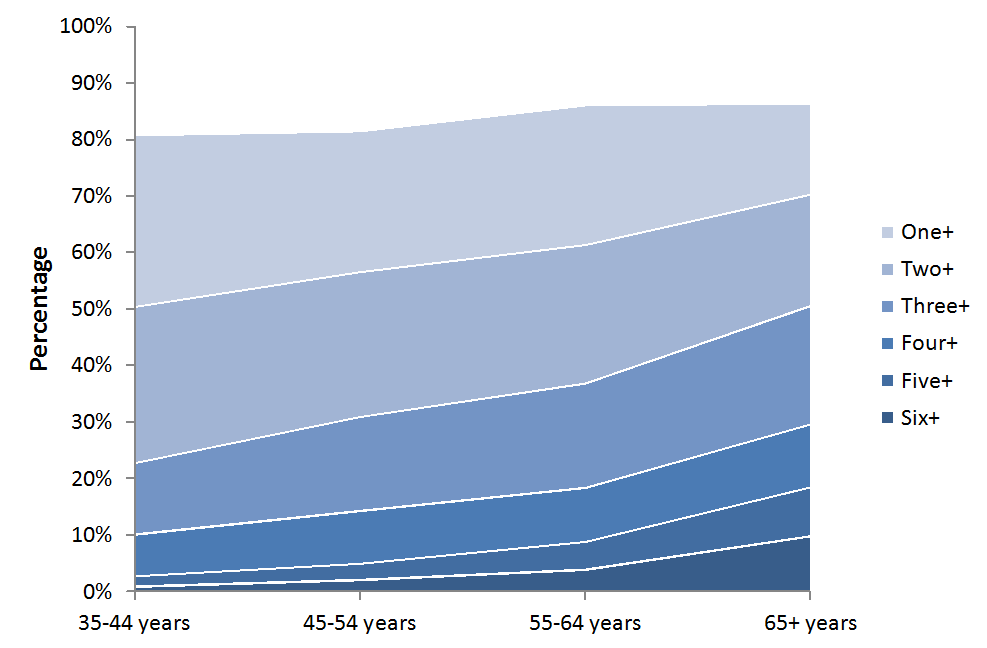


**Additional file 2: Figure S6**: percentage of participants with one or more condition(s), definition C.


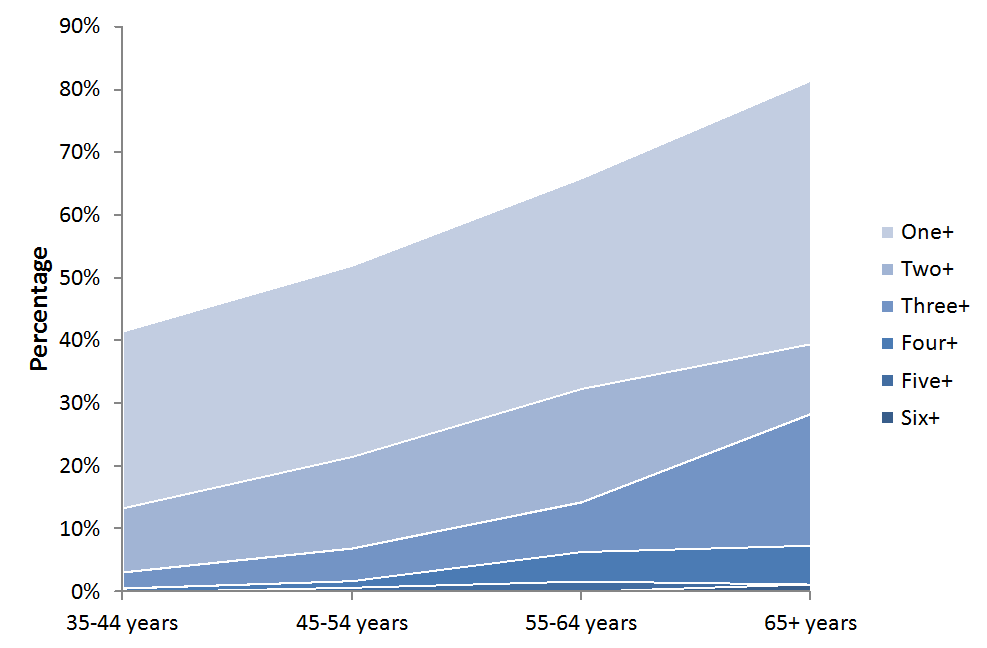

Supplement: Additional file 2: Figure S1. — Association between the number of reported and measured conditions. Figure S2. Association between the number of reported conditions and the number of conditions according to the Functional Comorbidity Index (FCI). Figure S3. Association between the number of measured conditions and the number of conditions according to the Functional Comorbidity Index (FCI). Figure S4. Percentage of participants with one or more condition(s), using self-reported conditions. Figure S5. Percentage of participants with one or more condition(s), using measured conditions. Figure S6. Percentage of participants with one or more condition(s), using Functional Comorbidity Index (FCI) criteria. [file 12889_2015_1515_MOESM2_ESM.docx]
